# Biotinylation as a tool to enhance the uptake of small molecules in Gram-negative bacteria

**DOI:** 10.1371/journal.pone.0260023

**Published:** 2021-11-12

**Authors:** Ankit Pandeya, Ling Yang, Olaniyi Alegun, Chamikara Karunasena, Chad Risko, Zhenyu Li, Yinan Wei

**Affiliations:** 1 Department of Chemistry, College of Arts and Sciences, University of Kentucky, Lexington, KY, United States of America; 2 Centre for Applied Energy and Research, University of Kentucky, Lexington, KY, United States of America; 3 Saha Cardiovascular Research Center, College of Medicine, University of Kentucky, Lexington, KY, United States of America; University of Cambridge, UNITED KINGDOM

## Abstract

Antibiotic resistance is a major public health concern. The shrinking selection of effective antibiotics and lack of new development is making the situation worse. Gram-negative bacteria more specifically pose serious threat because of their double layered cell envelope and effective efflux systems, which is a challenge for drugs to penetrate. One promising approach to breach this barrier is the “Trojan horse strategy”. In this technique, an antibiotic molecule is conjugated with a nutrient molecule that helps the antibiotic to enter the cell through dedicated transporters for the nutrient. Here, we explored the approach using biotin conjugation with a florescent molecule Atto565 to determine if biotinylation enhances accumulation. Biotin is an essential vitamin for bacteria and is obtained through either synthesis or uptake from the environment. We found that biotinylation enhanced accumulation of Atto565 in *E*. *coli*. However, the enhancement did not seem to be due to uptake through biotin transporters since the presence of free biotin had no observable impact on accumulation. Accumulated compound was mostly in the periplasm, as determined by cell fractionation studies. This was further confirmed through the observation that expression of streptavidin in the periplasm specifically enhanced the accumulation of biotinylated Atto565. This enhancement was not observed when streptavidin was expressed in the cytoplasm indicating no significant distribution of the compound inside the cytoplasm. Using gene knockout strains, plasmid complementation and mutagenesis studies we demonstrated that biotinylation made the compound a better passenger through OmpC, an outer membrane porin. Density functional theory (DFT)-based evaluation of the three-dimensional geometries showed that biotinylation did not directly stabilize the conformation of the compound to make it favorable for the entry through a pore. Further studies including molecular dynamics simulations are necessary to determine the possible mechanisms of enhanced accumulation of the biotinylated Atto565.

## Introduction

The ever growing antibiotic resistance in pathogens has become a serious global health threat and warrants a need for development of new therapeutics [1]. Among drug-resistant superbugs, gram-negative bacteria cause more serious concerns because of the dwindling pool of effective therapeutics. Their double layered cell envelope is difficult for antibiotics to penetrate. The asymmetrical outer membrane consists of lipopolysaccharides (LPS) and phospholipids in the outer and inner leaflet, respectively. Together with the phospholipid inner membrane, they make a tough barrier against both hydrophilic and hydrophobic antibiotics. Another major hurdle for antibiotics to cross the cellular envelope is the multiple efflux pumps that effectively transport many antibiotics out of cells [2]. Hence, there is an ongoing hunt for novel approaches to breach the barrier to increase the penetration and accumulation efficiency of antibiotics in gram-negative bacteria.

Several different approaches are being studied to address this penetration issue. Researchers are trying to establish the profiles that describe compound with good penetration behavior through the gram-negative cell envelope [3–5]. Retrospective study using the active antimicrobials as well as screening and computational algorithms have been successful in discovering some features shared by good penetrators. However, these rules are not holistic [6]. Apart from designing a potent inhibitor with optimal penetration features, an alternative approach that has been explored to address the impermeability issue is the “Trojan horse” technique. In this technique, the antibiotic is conjugated to a nutrient molecule and the uptake is driven by dedicated transporters of those nutrients present in the bacterial membrane [7]. The iron uptake system is such a system that has been the subject of study for several decades [8–12]. Siderophores are iron chelators released by bacteria to capture iron in the surrounding environment. Uptake of chelated iron is facilitated by the siderophore transporters. Antibacterial conjugated siderophores (sideromycins) occur in nature, for example albomycin, and are released by bacteria when they compete with other species for growing resources [13]. Mimicking nature, siderophore conjugates have been created for a couple of compounds to enhance their cellular accumulation through siderophore transporters. In some cases, the conjugates were cleaved once inside the cell to release the free drug [8,14,15]. Antibacterials made through this technique are showing promising results, especially the β-lactam-siderophore conjugates [14]. So far, the most promising candidate clinically is a siderophore antibiotic conjugate called Cefiderocol (S-649266), which is in phase 3 clinical trial [16]. This catechol cephalosporin conjugate has showed a promising antibacterial activity against a variety of Gram-negative pathogens including *Pseudomonas aeruginosa*, *Burkholderia cepacia*, *Acenitobacter baumannii*, *Enterobacteriaceae* and even the strains producing carbapenemases like *Klebsiella pneumoniae* carbapenemase and New Delhi metallo-β-lactamases (NDM)-1 [14,17].

Vitamin conjugation has also been explored to enhance drug penetration, mostly in mammalian cells, such as cancer cells. Since vitamins are critical for various metabolic reactions essential for survival, cells must either synthesize or uptake these vitamins from external environment through their transporters. Biotin (vitamin B7) mediated enhanced uptake has been shown to be effective in mammalian cell drug delivery, especially in cancer cells [18–20]. Sodium dependent multivitamin transporters (SMVT) are overexpressed in several different types of cancer cells, and it has been shown that biotin conjugation enhanced the uptake of anticancer molecule through these transporters [21,22]. Biotin conjugates are being studied because they can be both target-specific to cancer cells and can enhance the accumulation of drug through overexpressed biotin transporters [23].

However, in case of gram-negative bacteria, approaches involving biotinylation to enhance the uptake of small molecules have not been explored. Bacteria also require biotin for several metabolic processes, mainly the carboxylation and decarboxylation reactions. For this, bacteria either synthesize the biotin or uptake it whenever available through the biotin transporters [24]. In *E*. *coli*, there is only one major essential metabolic reaction carried out by Acetyl CoA carboxylase complex that needs biotin as a cofactor, although requirement in propionate metabolism have been reported in certain strains [25,26]. Acetyl CoA carboxylase acts in the conversion of acetyl CoA to malonyl CoA as in the fatty-acid biosynthesis process. The synthesis of biotin is such an energy expensive process that *E*. *coli* prefers to uptake biotin from the environment whenever available [25]. Biotin uptake in prokaryotes is believed to mostly occur through ATP dependent energy coupling factor (ECF) transporters, consisting of substrate specific S unit, transmembrane T unit, and ATPase containing A unit. In biotin transport, proteins BioY, BioN and BioM represent the S, T and A unit, respectively. However, in some bacteria the substrate specific BioY alone has been shown to transport biotin [27]. Recently it has been identified that in *E*. *coli*, biotin transport is mediated by a different protein called YgiM (BioP) through a yet not completely understood mechanism [24].

Walker et. al. [28] have shown that biotinylation facilitated the uptake of very large peptides up to 31 amino acids long that otherwise could not breach the membrane barrier of gram-negative bacteria. It was interesting to see that bacteria like *E*. *coli*, *Salmonella enterica* Typhimurium and *Pseudomonas aeruginosa* were able to uptake these large peptides through their biotin transporters [28]. Another example of exploiting nutrient uptake pathways for small molecules translocation was shown by Dumont et. al. where they demonstrated that the maltose porin LamB can transport and translocate the malto-triose conjugated fluorophores into the periplasm and cytoplasm of *E*. *coli* [29]. Recently, one study by Zhao et. al. [30]. focused on vitamin B12 mediated enhanced uptake where they showed ampicillin upon conjugation with vitamin B12 had about 500 folds improved efficacy than the parent ampicillin. They demonstrated that this increased efficacy is due to the enhanced uptake through vitamin B12 transporters [30].

However, besides biotinylation mediated peptide uptake, to our knowledge there are no such studies that focused on the biotinylation mediated uptake of small molecules in gram-negative bacteria. Thus, we tried to probe how biotinylation will affect the penetration and accumulation of small molecules using florescent compound Atto565 and *E*. *coli* as a gram-negative model.

## Materials and methods

### Bacterial strains and chemicals

Atto565, Atto565-biotin, Atto565-NHS and all the amino acids used were obtained from Sigma-Aldrich (St. Louis, MO). Bacterial strain BW25113 was obtained from Yale E. coli Genetic Stock Centre, BL21(DE3) strain was obtained from New England Biolabs (Ipswich, MA) and MG1655WT, ΔOmpF, ΔOmpC and ΔOmpF&C were kindly provided as a gift by Dr. Chang-Ro Lee (Department of Biological Sciences and Bioinformatics, Myongji University, Yongin, South Korea).

### Compound accumulation assay

Overnight bacterial culture was diluted 50 folds in fresh and sterile LB and cultured at 37 degree Celsius with constant shaking at 250 rpm until they reach mid-log phase (OD ~0.6). After that, the cells were harvested through centrifugation at 3000×g for 15 minutes, washed once using the NaPi-Mg buffer (50 mM sodium phosphate buffer with 5 mM MgCl_2_, pH 7.4) and then resuspended in the same buffer to a final concentration of OD_600nm_ 8.0. From this cell suspension, 2 mL was taken in a glass vial and 250 μL of compound (10X) was added and final volume was adjusted to 2.5 mL using the buffer to make the final concentration of compound 2 μM and final OD_600nm_ 6.4. The mixture was then incubated for 30 minutes with constant shaking at 250rpm at 28 degree Celsius. After the incubation, 700 μL of suspension was taken and carefully layered on top of 700 μL silicone oil (AR20: Sigma High temperature = 9:1) [3] in a microcentrifuge tube. It was then centrifuged for 1 minute at 13,000g and the supernatant composed of upper aqueous layer and lower oil layer was carefully removed using micropipette. Kim wipes were then used to wipe the inner wall of the tube and the pellet was resuspended in 1 mL of the lysis buffer (50mM Tris pH 8.0, 0.5% Triton-X100, 0.5% glycerol, 100 μg/mL lysozyme, 10 μg/mL DNase). Next, samples were subjected to 6 cycles of freeze and thaw, followed by centrifugation for 15 minutes at 15,000×g to remove the cell debris. The supernatant was collected to measure the florescence. For both Atto565 and Atto565-biotin, excitation wavelength used was 565 nm and the emission was monitored from 575 nm to 620 nm, and the emission maxima as 585 nm were recorded. All the measurements were done in triplicate and the data were presented as average ± standard deviation.

### Fractionation of periplasm and cytoplasm

Periplasm and cytoplasm were separated according to the osmotic shock method we described previously [31]. Briefly, the pellet obtained after incubation with compounds and centrifugation was resuspended in 100 μL periplasm preparation buffer (200 mM Tris-Cl, 1 mM EDTA, 20% sucrose, 1 mg/mL lysozyme, pH 8.0). The resuspension was then incubated at room temperature for 5 minutes, followed by the addition of 200 μL ice cold deionized water. The mixture was mixed properly by tapping or pipetting and was incubated on ice for 2 minutes. Samples were then centrifuged for 1 minute at 13,000×g. The supernatant contained the periplasmic components, and the pellet contained the spheroplast. Spheroplast was further lysed according to the procedure as mentioned above to release the cytoplasmic components.

### Expression of streptavidin and biotin-streptavidin binding

Streptavidin was expressed using the pET30b vector. pET30b_OmpAT7Sav (Addgene #138589) [32] was used to express streptavidin in the periplasm, and pET30b_T7Sav (Addgene #138588) [25] was used to express streptavidin in the cytoplasm of BL21(DE3) cells. Biotin is essential for cell growth as it is a cofactor for the enzyme Acetyl CoA carboxylase that converts acetyl CoA into Malonyl CoA needed for fatty acid biosynthesis. Expression of streptavidin inside the cell is toxic as it will bind to the biotin and deprive biotin for fatty acid biosynthesis. To circumvent this detrimental effect, streptavidin was expressed in conjunction with MatC transporter and MatB enzyme from the plasmid pCKmatBC (Addgene #138587) [25], which transport malonate from the media (MatC) and convert it into malonyl CoA (MatB). Through this strategy, the need for biotin in the conversion of acetyl CoA to malonyl CoA was bypassed.

The expression of streptavidin was carried out by inducing the mid log phase cells with 0.5 mM IPTG for 45 minutes and then accumulation experiment was done as described in section 2.2. Binding to streptavidin quenches the fluorescence of biotinylated Atto565. To dissociate Atto565-biotin from streptavidin, a very high concentration (3 mM) of free biotin was added into the cell lysate and samples were subjected to a heat treatment of 78 degree Celsius for 10 minutes. Next the samples were cooled down, centrifuged for 15 minutes at 15,000×g, and then the supernatant was collected for fluorescence measurement. High temperature helped to break the biotin/streptavidin interaction, and the presence of high concentration of free biotin helped to saturate the binding site of streptavidin to free Atto565-biotin.

### Cloning and expression of OmpC

The OmpC gene was amplified using PCR from the genomic DNA of *E*. *coli* MG1655WT strain and it was cloned into plasmid pACYCT2 (Addgene #45799) [33] using the “Fast cloning” method [34]. The OmpC encoding plasmid, pACYCT2-OmpC, was then transformed into the *E*. *coli* MG1655*ΔompC* strain. Fresh transformants were picked and cultured overnight in LB with 25 μg/ml chloramphenicol. This overnight culture was used to inoculate a fresh LB/Chloramphenicol media. OmpC expression was induced when the culture grew to the mid log phase with 1 mM IPTG for 30 mins at 28 degree Celsius. Cells were then harvested and subjected to accumulation experiment as described in section 2.2. For the visualization of OmpC expression, 1 mL of OD 8.0 cells were lysed through sonication (50% amplitude, 1 min, 5 secs on/off) and centrifuged for 15 minutes at 15,000×g to get the membrane fraction. Supernatant was discarded and the membrane pellet was resuspended in 50 μL NaPi-Mg buffer, 75 μL 10% SDS and 25 μL of 6X SDS loading dye. The mixture was incubated for 1 hour at room temperature for protein extraction. After that, the samples were boiled for 10 minutes and visualized through SDS-PAGE in 15% polyacrylamide gel.

### Site directed mutagenesis and knockouts

Site directed mutagenesis was done using the QuikChange Site Directed Mutagenesis Kit (Agilent, Santa Clara, CA) following the manufacturer’s protocols. Primers for the introduction of D18V, W72K and D171T mutations are shown in S1 Table. *LamB* knockout strain was created using the Quick & easy *E*. *coli* gene deletion kit (Gene Bridges GmbH, Heidelberg, Germany) following the manufacturer’s protocol. The target gene on the MG1655 chromosome was replaced with an FRT-flanked kanamycin resistance marker cassette. Colony PCR was performed to confirm that the target gene has been replaced correctly. All constructs were confirmed through sequencing.

### Preparation of amino acids labelled Atto565

For the preparation of amino acids labelled Atto565, we used the Atto565-NHS ester and used it to react with amino groups of amino acids using the same principle of protein labeling by NHS ester of a compound. Labeling was done using 20 μM Atto565-NHS and 50 mM amino acids in a 200 mM Na-Pi buffer, pH 8.0 for 2 hours at room temperature. The resulting labeled solution was then used for the accumulation experiment. Seven different amino acids (Glycine, Arginine, Lysine, Aspartic acid, Glutamic acid, Asparagine, Glutamine) were used including representatives of acidic, basic, and neutral amino acids.

### Density functional theory (DFT) calculations

Density functional theory (DFT) calculations were carried out at the ωB97XD/6-31G(d,p) level of theory to optimize molecular geometries, and charges were explicitly determined using the Charge Model 5. The Polarizable Continuum Model (PCM) with the integral equation formalism variant (IEFPCM) was used to simulate water solvent reaction field. The multi-wfn [35,36] package was used for the generation of Hirshfield charge distributions [37] for the DFT level energy optimized structures. All visualizations were carried out using Visual Molecular Dynamics (VMD) package version 1.9.3 [38] with colors red and blue representing the atomic charge range of -0.5 and +0.5 (a.u.) respectively.

### Statistical analysis

All the data shown were represented as mean±S.D. Significance was analyzed by either One-way or Two-way analysis of variance (ANOVA). Specific details about the asterisks presented above the bar or group of bars and significance measurements are presented in the figure legends.

## Results

### Atto565-biotin accumulates higher in *E*. *coli* than the Atto565

**Fig 1:**
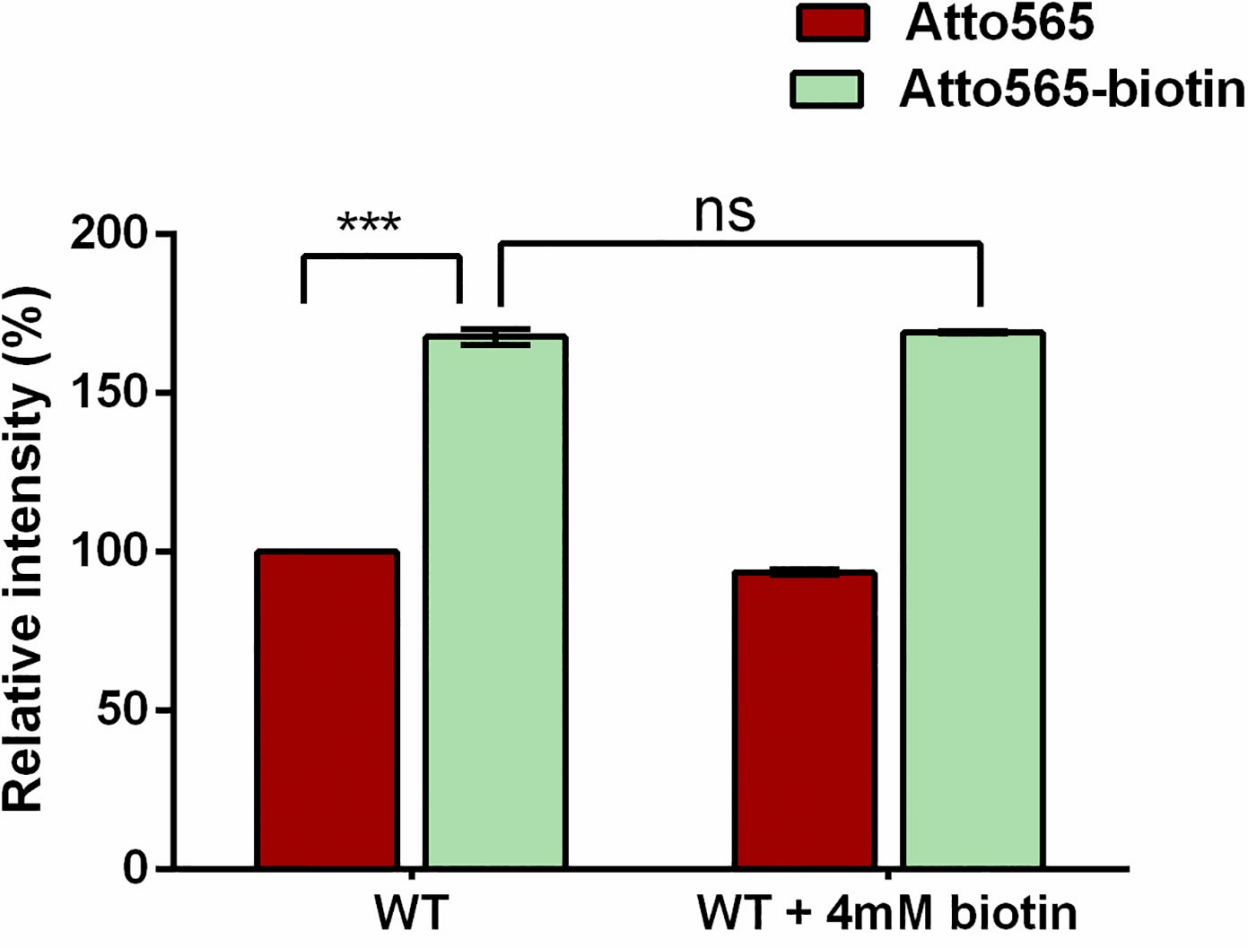
**Accumulation of Atto565 (red) and Atto565-biotin (green) in *E*. *coli*, in the presence or absence of free biotin.** Figure shows representative data from three independent experiments. Data were presented as mean±S.D. (Two-way analysis of variance; ***p<0.001).

To determine whether biotinylation of compounds would enhance their accumulation in gram-negative bacterial cells, we first measured the accumulation of a florescent compound, Atto565, and its biotinylated form, Atto565-biotin, in *E*. *coli*. The cells were incubated with the compound and then harvested. After cell lysis and centrifugation, florescence signal in the supernatant was monitored. We observed that Atto565-biotin accumulated at a significantly higher level compared to the parent compound Atto565 (Fig 1). To determine if the higher accumulation is because of the uptake through biotin transporter, we did a competitive experiment by adding an excess of free biotin (4 mM) in the presence of Atto565-biotin (2 μM) or Atto565 (2 μM) during the incubation. If the uptake was through biotin transporters, the presence of a high concentration of free biotin would compete and impair the uptake of Atto565-biotin but not Atto565. However, we did not see a competitive effect from the 2000-fold excess of free biotin (Fig 1). The accumulation of Atto565-biotin was very similar with or without the presence of free biotin in the medium, indicating that biotin transporters are not involved in the enhanced uptake of Atto565-biotin.

### Biotinylation enhances the penetration of Atto565 across the outer membrane (OM)

**Fig 2:**
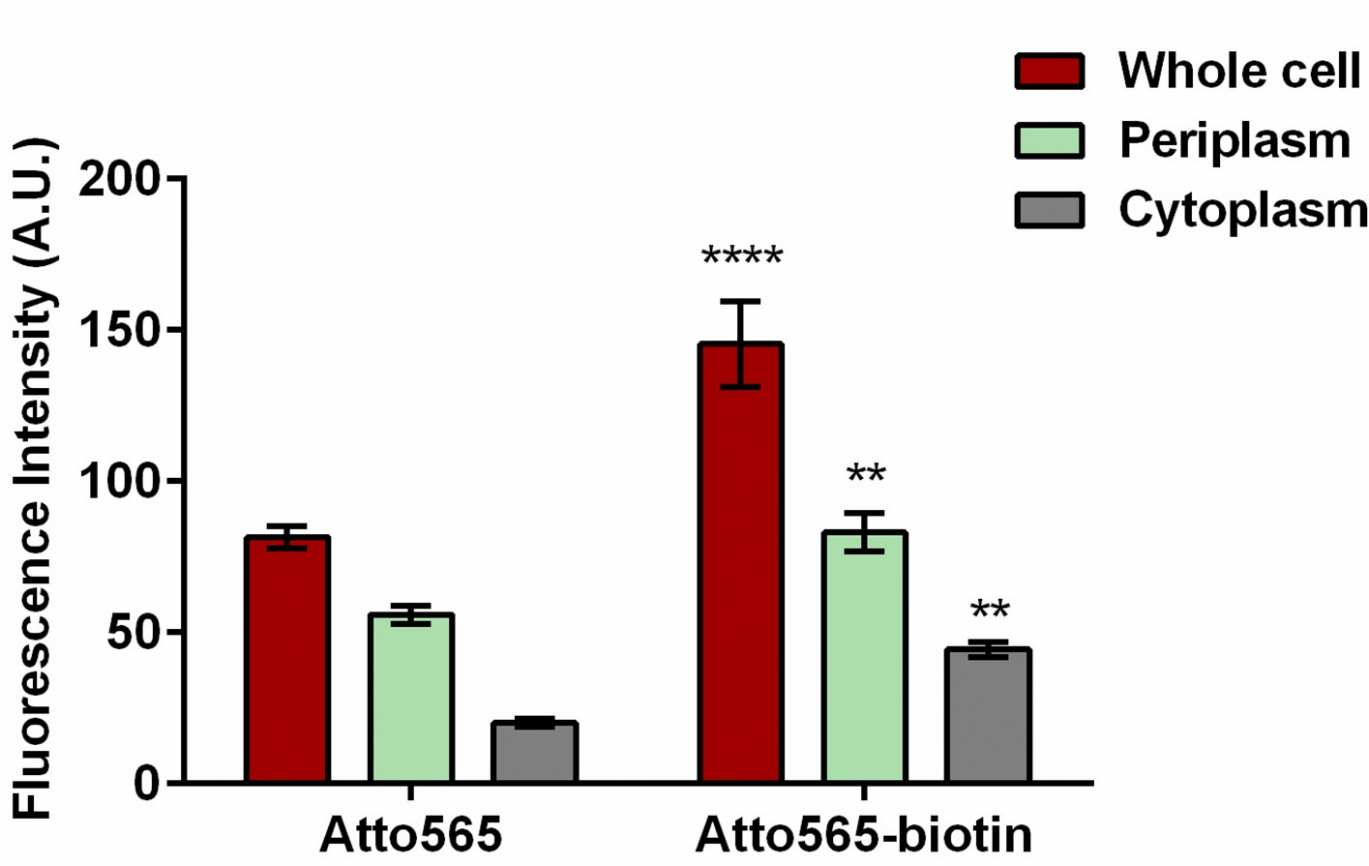
Subcellular accumulation of Atto565 and Atto565-biotin. Figure shows representative data from three independent experiments. Data were presented as mean±S.D (Two-way analysis of variance; ****p<0.0001, **p<0.01) Asterisk represents the significance when compared Atto565-biotin accumulation with Atto565 accumulation in respective subcellular compartment.

Next, we measured the subcellular distribution of the compounds in the periplasm and cytoplasm to determine whether biotinylation promotes uptake of Atto565 through breaching the OM or the IM. We separated the periplasm from the cytoplasm and measure the fluorescence intensities in each sample. We found that the compounds were mostly located in the periplasmic fraction and a small portion of the compounds was in the cytoplasmic fraction (Fig 2).

**Fig 3:**
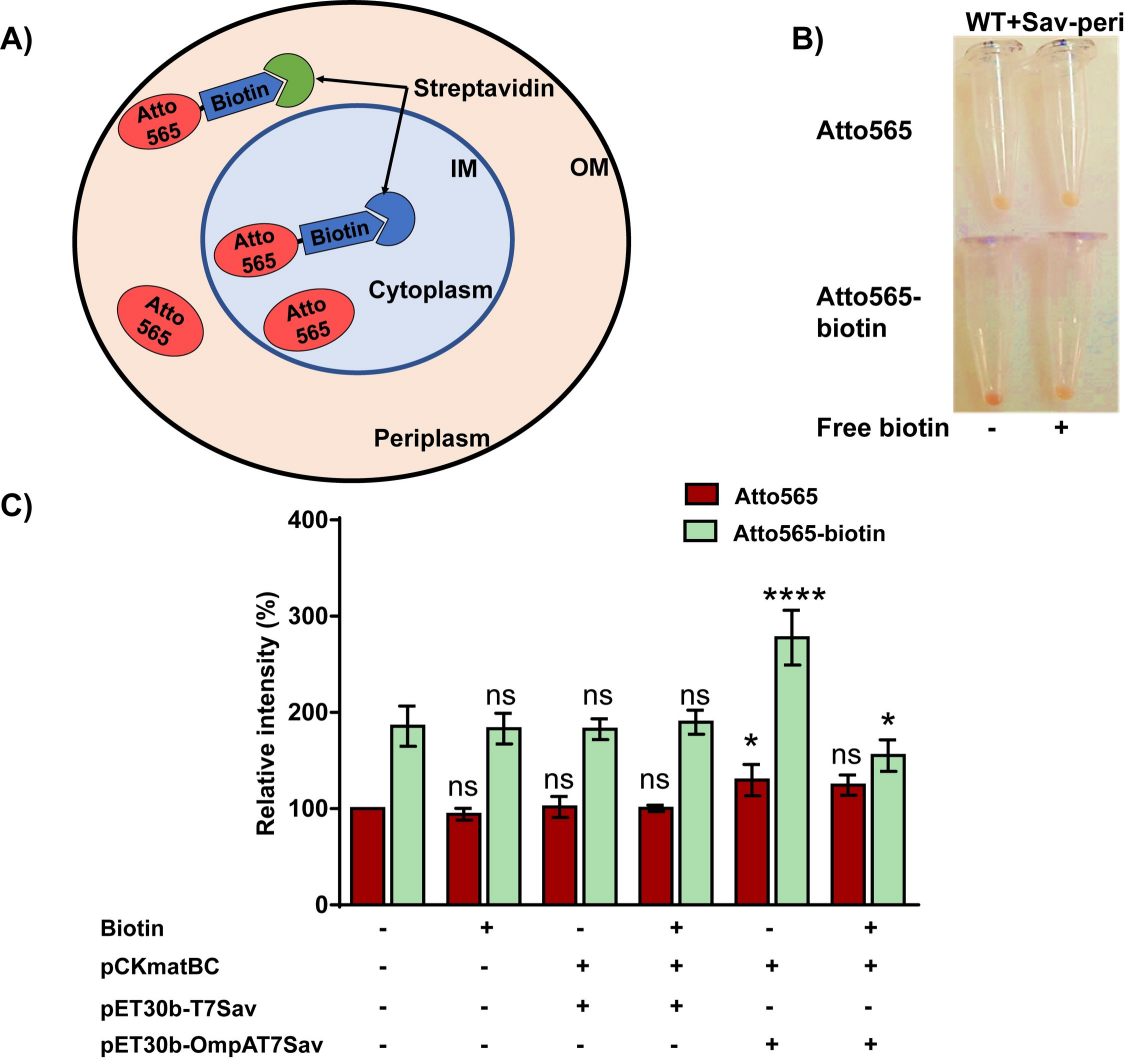
Accumulation of Atto565 and Atto565-biotin in *E*. *coli* expressing streptavidin either in the periplasm or cytoplasm. A) Schematics showing plasmid-expressed streptavidin binds to Atto565-biotin. B) Image of cell pellets showing redder color (higher accumulation) of Atto565-biotin in cells expressing periplasmic streptavidin and decrease in the accumulation in the presence of free biotin. C) Quantitative analyses of accumulation of Atto565 and Atto565-biotin in cells expressing streptavidin in the presence or absence of free biotin. Figure shows the representative data from three independent experiments. Data were presented as mean±S.D. (Two-way analysis of variance; ****p<0.0001, *p<0.05). Asterisks above the Atto565 accumulation bar represent significance when compared them with Atto565 accumulation in WT strain, whereas asterisk above the Atto565-biotin accumulation bar represent the significance when compared with Atto565-biotin accumulation in WT strain.

To verify that Atto565 entered the cells, we measured accumulation of the fluorescent compound in *E*. *coli* strains expressing streptavidin. We expressed streptavidin in the periplasm or cytoplasm using plasmids pET30b_OmpAT7Sav and pET30b_T7Sav, respectively, as described in materials and method. We expect that if biotinylation really enhanced cellular accumulation of Atto565, a higher accumulation would be observed in strains expressing streptavidin. As a control, we also examined the impact of streptavidin expression on the accumulation of Atto565. We found that when streptavidin was expressed in the periplasm, the accumulation of Atto565-biotin increased but the accumulation of Atto565 remained unchanged (Fig 3). When free biotin was present in large excess, the accumulation of Atto565-biotin decreased. This clearly indicated that Atto565-biotin entered the periplasm. In contrast, when streptavidin was expressed in the cytoplasm of the cell, the accumulation of Atto565-biotin did not increase (Fig 3).

### Biotinylation does not affect efflux

**Fig 4:**
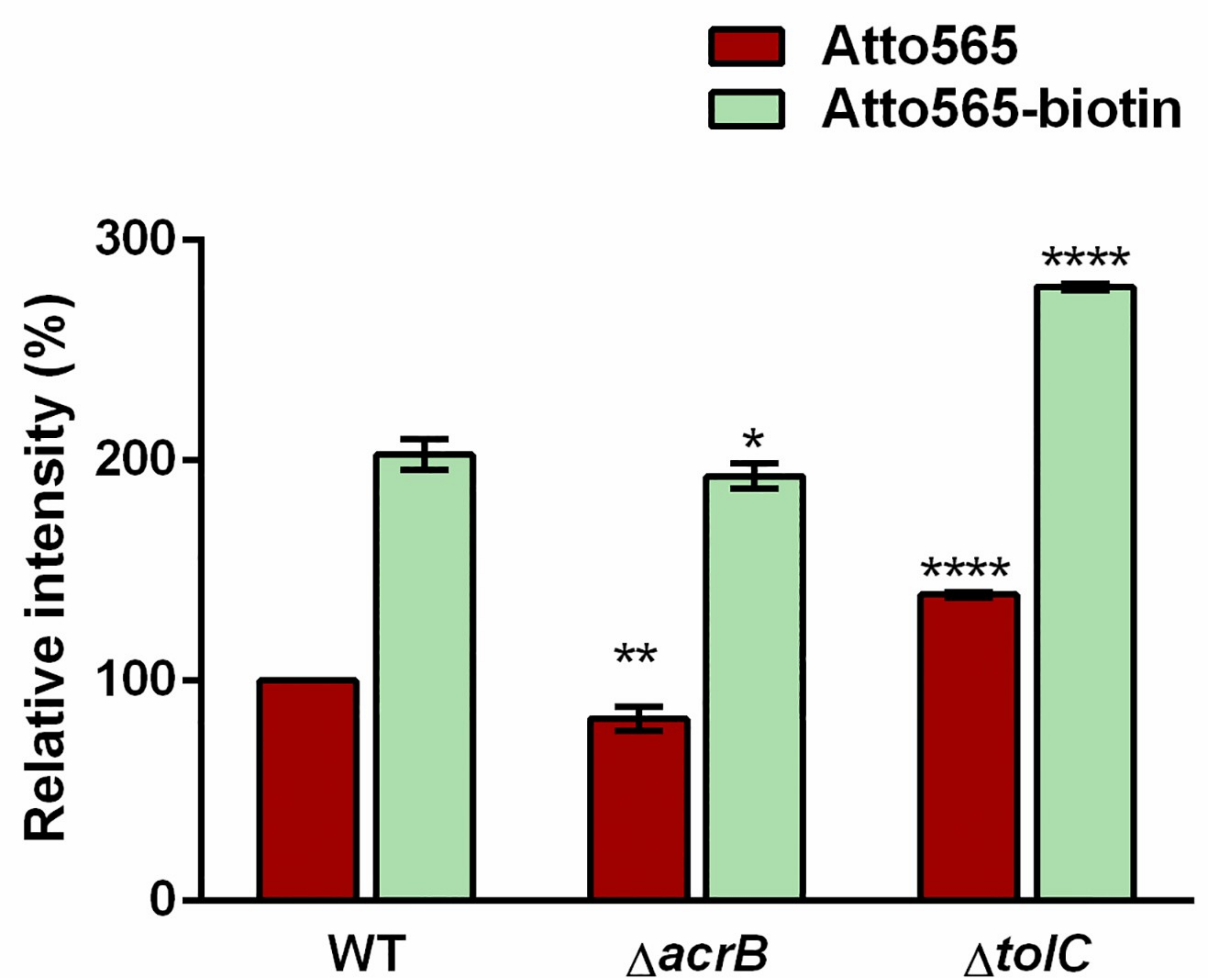
Accumulation of Atto565 and Atto565-biotin in wild type versus efflux knock out BW25113 strains. Figure shows the representative data from three independent experiments. Data were presented as mean±S.D. (Two-way analysis of variance; ****p<0.0001, **p<0.01, *p<0.05). Asterisks above the Atto565 accumulation bar represent significance when compared them with Atto565 accumulation in WT strain, whereas asterisk above the Atto565-biotin accumulation bar represent the significance when compared with Atto565-biotin accumulation in WT strain.

Another possible factor that may lead to the observed higher accumulation of Atto565-biotin than Atto565 is efflux. If biotinylation makes a compound a bad substrate for efflux pumps, then higher accumulation would also be observed. We tested the efflux efficiency of both compounds by doing the accumulation experiment in wild type as well as in a deficient strain lacking major efflux systems. We used BW25113 and its isogenic *ΔacrB* and *ΔtolC* strains. Deletion of the *acrB* gene did not have a significant effect on accumulation of both compounds. A higher accumulation for both compounds were observed in the *ΔtolC* strain than in the WT strain, indicating that they both are substrates of efflux involving TolC [Fig 4]. The ratio of accumulation of Atto565 between the WT strain and *ΔtolC* strain was very similar to the ratio of accumulation of Atto565-biotin between the WT and *ΔtolC* strain, suggesting that the extent of efflux was similar for both compounds. Thus, higher accumulation of Atto565-biotin is not because it is a bad substrate for TolC-involved efflux. Also, since we know efflux pumps can pump out substrates from either periplasm, inner membrane or cytoplasm [39], the observation that both of these compounds were substrate of efflux pumps further verified that the compounds entered the cells, not just attached to the cell surface.

### OmpC contributes to the elevated accumulation of Atto565-biotin

**Fig 5:**
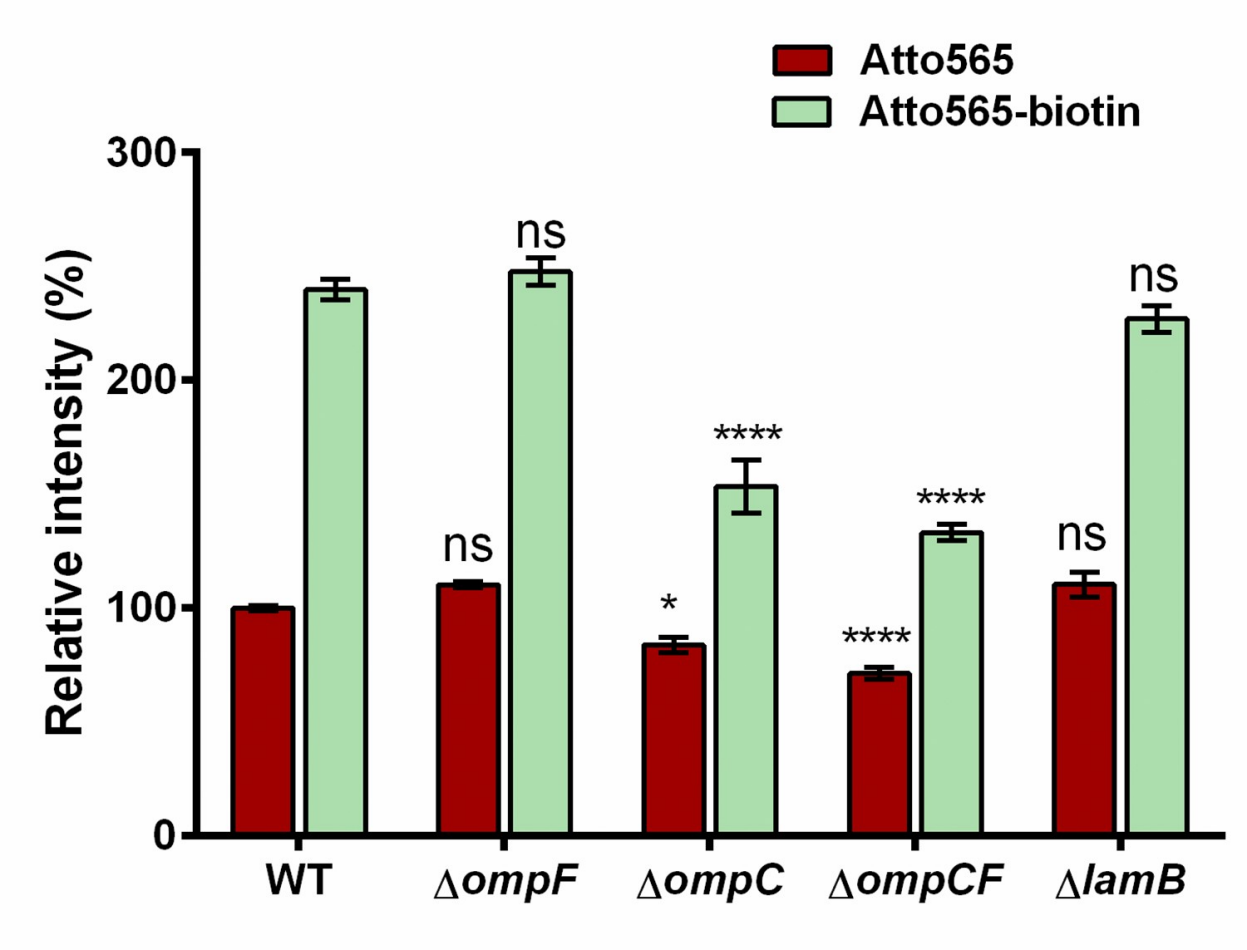
Accumulation of Atto565 and Atto565-biotin in wild type versus porin knock out strains. Figure shows the representative data from three independent experiments. Data were presented as mean±S.D. (Two-way analysis of variance; ****p<0.0001, *p<0.05). Asterisks above the Atto565 accumulation bar represent significance when compared them with Atto565 accumulation in WT strain, whereas asterisk above the Atto565-biotin accumulation bar represent the significance when compared with Atto565-biotin accumulation in WT strain.

Generally, compounds cross the outer membrane through porins, lipid mediated diffusion, or self-promoted uptake [40]. We performed the accumulation assay using MG1655 and various isogenic porin knockout strains. We found that the accumulation of Atto565-biotin was greatly reduced in MG1655Δ*ompC* where the reduction of accumulation of parent compound was minimal [Fig 5]. This reduction was not observed in MG1655Δ*ompF*, which showed similar accumulation as the wild-type strain. Also, MG1655Δ*ompFΔompC* double knockout strain showed similar accumulation as the MG1655Δ*ompC* single knock out strain. Another major porin apart from OmpF and OmpC is the sugar porin LamB, which is regarded mostly as the pathway for sugar transport including maltose and maltodextrins. We also tested the accumulation in the strain lacking this sugar porin LamB. As expected, the accumulation was similar as the wild-type strain, ruling out the involvement of LamB in higher accumulation of Atto565 after biotinylation. This indicated that OmpC serves as a port of entry for Atto565-biotin. Biotinylation makes the Atto565 a better passenger through OmpC.

**Fig 6:**
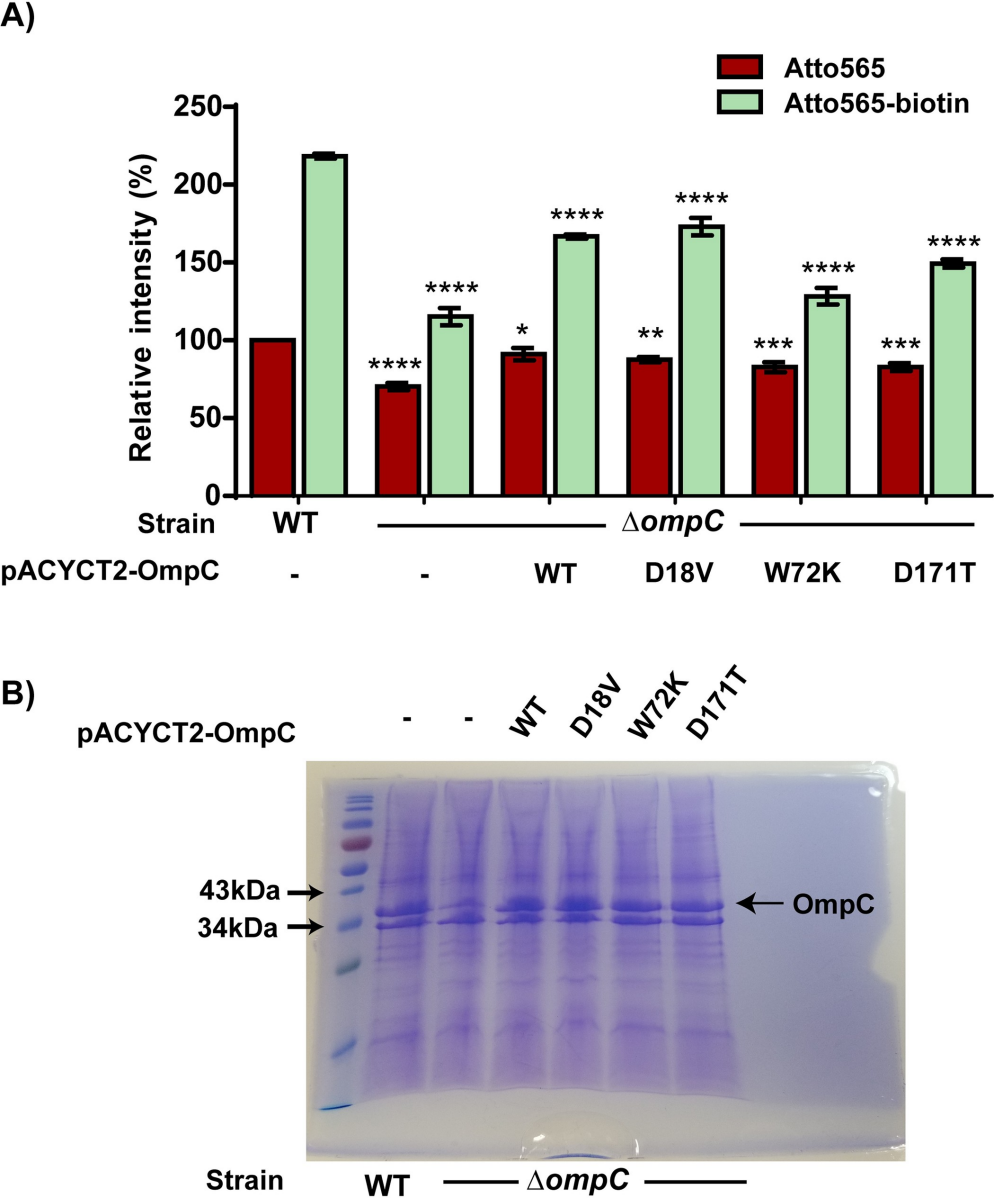
Accumulation of Atto565 and Atto565-biotin upon plasmid complementation of wild type OmpC as well as OmpC with different single mutations. Figure shows the representative data from three independent experiments. Data were represented as mean±S.D. (Two-way analysis of variance; *p<0.05, **p<0.01, ***p<0.001, ****p<0.0001). Asterisks above the Atto565 accumulation bar represent significance when compared them with Atto565 accumulation in WT strain, whereas asterisk above the Atto565-biotin accumulation bar represent the significance when compared with Atto565-biotin accumulation in WT strain.

To further verify the role of OmpC, we complemented the *BW25113ΔompC* strain with plasmid pACYCT2-OmpC, which encodes the *ompC* gene under the control of the *tac* promoter and IPTG inducible *lac* operator. Upon induction of OmpC expression from the plasmid, accumulation of Atto565-biotin was partial restored in the *BW25113ΔompC* strain (Fig 6).

Both OmpC and OmpF are major porins in *E*. *coli* and favor cationic molecules, but OmpC has a higher preference to cations than OmpF [41–43]. The sequences of the two proteins are highly homologous, with 59.5% identity and 72.1% similarity. A few different ionizable amino acid residues lining the pore lumen have been proposed to be involved in making interactions with substrates during their passage through the channel [43]. We replaced these residues in OmpC with their corresponding residues in OmpF and tested the impact of these mutations (D18V, W72K and D171T) on the accumulation of Atto565 and Atto565-biotin (Fig 6). We found that the W72K mutation reduced the accumulation the most. When the tryptophan was converted into a lysine, expression of the mutant did not restore accumulation to the ompC knock out strain. In contrast, the D18V mutation did not seem to affect passage of Atto565-biotin. Expression of the D171T mutant partially restored accumulation, at a level in between the levels in cells expressing W72K (close to the *ompC* knockout strain) and D18V (similar to the *ompC* knockout strain supplemented with plasmid encoded OmpC). We examined the expression levels of these mutants and confirmed that the mutants expressed at a similar level as the wild type OmpC (Fig 6). The observed differences in accumulation are not due to variation in protein expression.

### Effect of labels other than biotin in accumulation of Atto565

**Fig 7:**
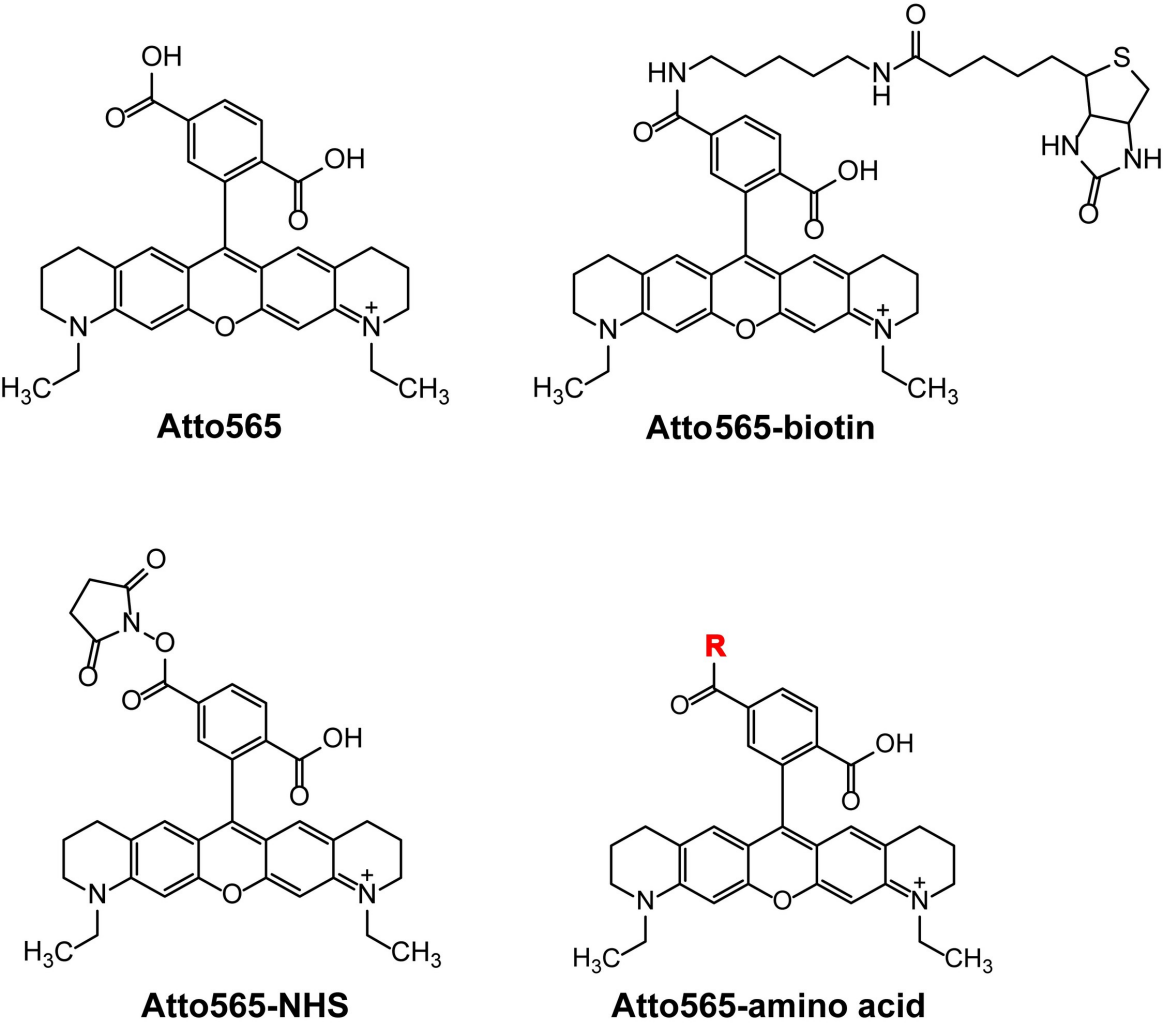
Chemical structures of Atto565, Atto565-biotin, Atto565-NHS ester and the Atto565 labelled with various amino acids (Glycine, Lysine, Arginine, Aspartic acid, Glutamic acid, Asparagine, Glutamine) represented by -R group.

The structure of Atto565 contains one ionizable amine group and two carboxylic acid groups, so at neutral pH the molecule will have a net negative charge. In Atto565-biotin, one of the carboxylic acid group is altered to form an amide bond to connect with biotin, thereby eliminating one negative charge. We speculated that the elimination of one negative charge could make it a more favorable passenger through the OmpC channel as OmpC prefers cations [42,43]. To elucidate the role of eliminating a negative charge, we reacted Atto565-NHS ester with various neutral (glycine, glutamine, and asparagine), positively charged (lysine and arginine), and negatively charged amino acids (aspartic acid and glutamic acid) (Fig 7).

**Fig 8:**
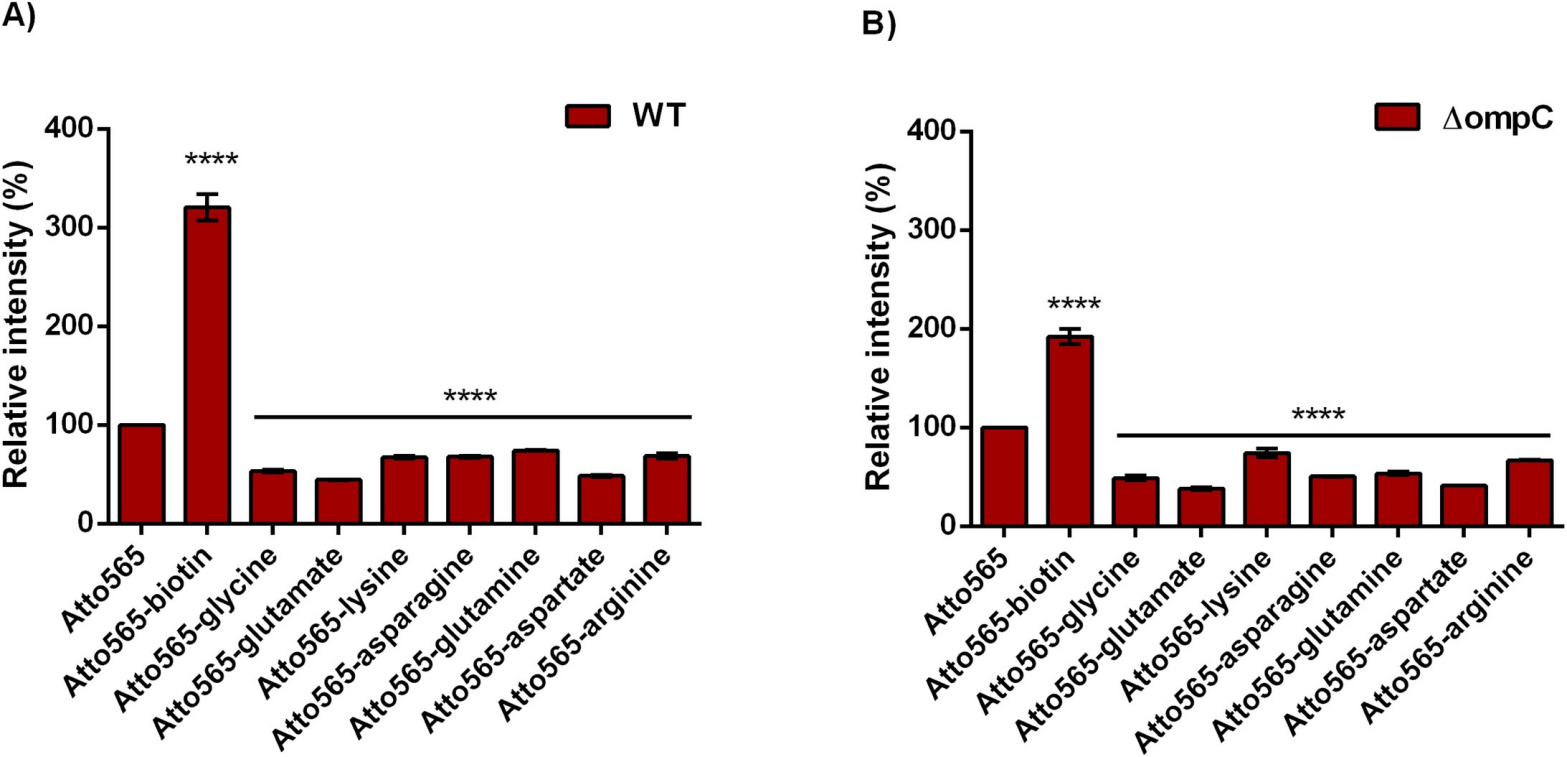
Accumulation of different amino acids labelled Atto565 in wild type (A) and ΔOmpC (B) strains. Data were presented as mean±S.D. (One-way analysis of variance; ****p<0.0001). Asterisks above the bars represent the significance compared with the accumulation of Atto565.

Accumulation of Atto565-amino acid derivatives are shown in Fig 8. Florescence was also measured for each of the labelled compound to control for the potential effect of the labeling on fluorescence intensity [S1 and S2 Figs] When the florescent intensities were normalized using the calibration curves of each compound, it was observed that in general all Atto565 conjugates accumulated less than the parent compound Atto565. Among the conjugate compounds, negatively charged amino acids (glutamic acid and aspartic acid) reduced accumulation further than neutral and positively charged amino acids. This is consistent with the know property of OmpC for preferring positive charges.

### Molecular charge distributions and geometries

**Fig 9:**
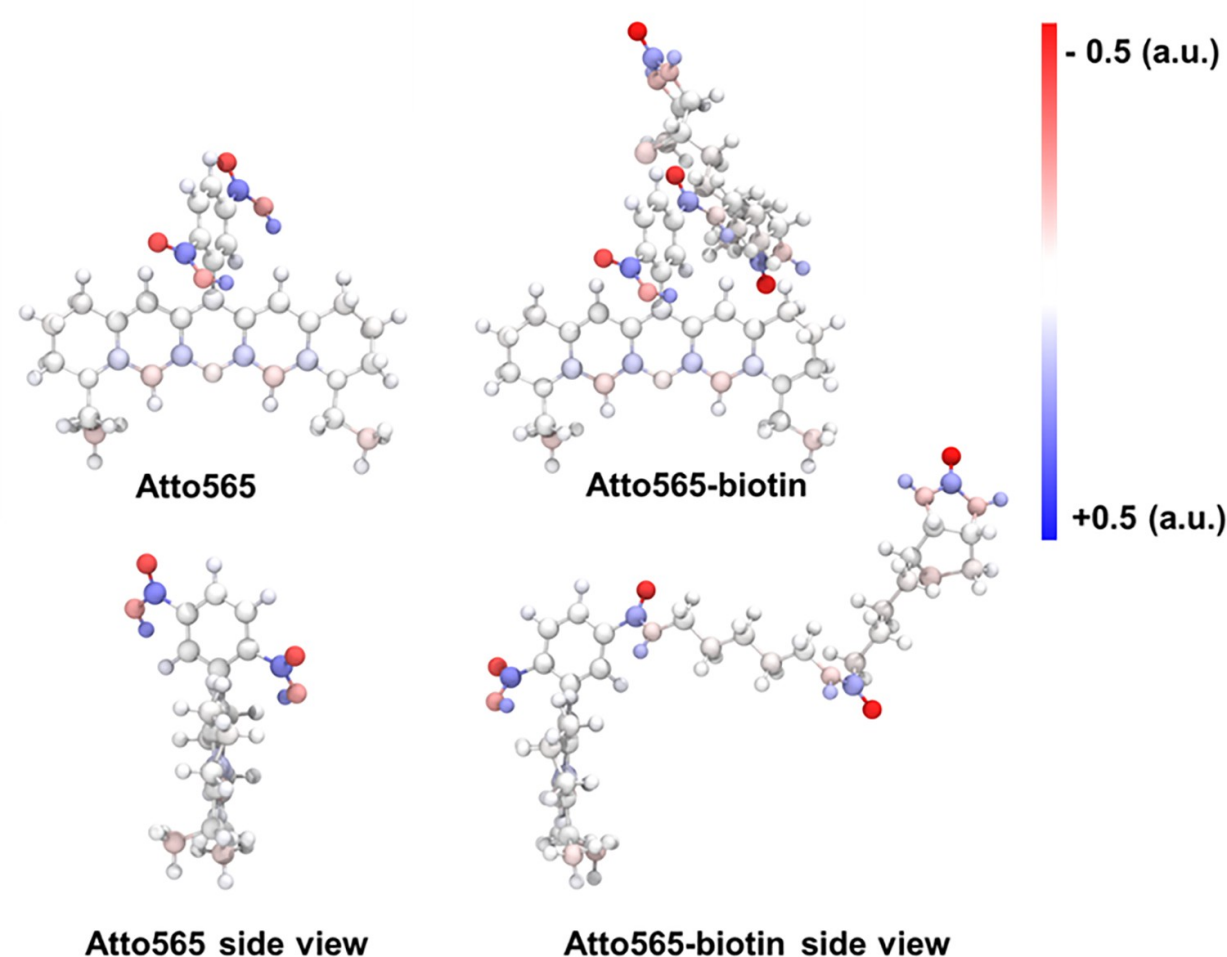
Lowest enegry conformations of the DFT-minimized structures. The red and blue colors represent the atomic charge range of -0.5 and +0.5 respectively.

To further probe the potential impact of biotinylation on Atto565, we examined the Hirshfield charge distributions and energy optimized geometries as determined by DFT calculations at the ωB97XD/6-31G(d,p) level of theory. All structures showed similar charge distribution along the π-conjugated backbone, with the extra positive charge delocalized between the amides. The DFT-optimized molecular geometries, as shown in Fig 9, reveal no real structural difference of the Atto565 in either the biotinylated or non-biotinylated forms. We note, however, that these are static pictures, and that molecular dynamics in the cellular environment could demonstrate variations in conformational flexibility. Further, the alkyl chains with functional groups could be of importance for establishing non-covalent interactions with OmpC to accommodate cellular penetration.

## Discussion

Protection from the outer membrane and the inner membrane, as well as the presence of efflux pumps make the gram-negative bacterial envelope a formidable barrier for compounds to penetrate. This is becoming a significant hurdle in the development of new antimicrobials. Small hydrophilic molecules generally cross the outer membrane through porin pores and hydrophobic molecules pass through passive diffusion, which is also not easy because of the presence of lipopolysaccharides outside the outer membrane. In addition, the inner membrane restricts penetration of small hydrophilic molecules. Several approaches are being developed to solve this issue. Conjugation of antimicrobials with nutrient molecules to facilitate their uptake through the nutrient uptake systems is such an approach.

In this study we examined the concept of using biotin to assist efficient penetration of selected compound into Gram-negative membrane. Based on our results, we found that biotin conjugation helps in the increased penetration of florescent molecules. However, in contrary to our expectation, the increased penetration did not seem to occur through biotin-specific uptake. When we measured the subcellular distribution of the accumulated compounds, it was observed that the majority of the compounds were accumulated in the periplasmic space. To determine if biotinylation actually promoted accumulation of atto565 inside of cells, we examined the effect of expressing streptavidin. Our result clearly showed that expression of streptavidin in the periplasm specifically increased accumulation of Atto565-biotin, which was repressed in the presence of excess free biotin. This observation indicates that Atto565-biotin did enter the periplasm and was trapped by streptavidin. However, upon cytoplasmic expression of streptavidin the accumulation of Atto565-biotin did not increase significantly. Biotinylation of Atto565 seemed to promote penetration across the outer membrane but not the inner membrane. Further experiment using efflux knockout strains further confirmed that the compound did enter the cells, as non-specific cell surface attachment would have similar effect in different efflux knockout strains. We used *acrB* and *tolC* gene knockout strains to evaluate the role of efflux in the accumulation of these compounds. AcrABTolC system is the resistance nodulation division (RND) superfamily type efflux pump which is a major contributor of the drug efflux in *E*. *coli*. AcrB is the inner membrane pump, AcrA is the periplasmic adapter and TolC is the outer membrane channel in this system [44]. However, TolC can also be coupled with several other efflux pump systems in *E*. *coli*. [45]. We observed higher accumulation of both compounds in *tolC* deficient strain, indicating that the efflux system involving TolC could pump out Atto565 and Atto565-biotin. In contrast, deletion of the *acrB* gene did not have such an effect, indicating that TolC worked with another inner membrane protein/adaptor protein pair to efflux Atto565 and Atto565-Biotin.

If not through biotin-specific transporters, how did biotinylation improve accumulation? We found that the pathway of entry involves OmpC, but not a closely related porin OmpF. OmpC deficient strain accumulated less Atto565-biotin, while complementation with plasmid-encoded OmpC partially restored accumulation. Although the restoration was not 100%, the increase was significant. Also, single residue mutations introduced to replace ionizable amino acids in the pore lining of OmpC affected Atto565 accumulation, further confirming the role of OmpC as a route of entry. This evidence supported that the Atto565-biotin permeated *E*. *coli* outer membrane better than Atto565, and that this enhanced penetration is at least partially through the OmpC porin. This enhanced penetration through the outer membrane could be of great importance as the outer membrane is a critical barrier of defense for Gram-negative bacteria. There are several antibiotics which are effective against gram-positive bacteria but not against gram-negative bacteria, because of this extra outer membrane and active efflux [3,4]. So, development of methodologies that can help the otherwise impermeable molecules to pass through the outer membrane is beneficial to design antibiotics effective against Gram-negatives. Subcellular distribution studies and the streptavidin expressing experiments revealed that the accumulation of both compounds was not very significant in the cytoplasm and biotinylation only increased the cytoplasmic accumulation slightly. Biotinylation did not seemed to promote Atto565 permeability across the inner membrane.

OmpC is considered a non-specific porin present in the outer membrane of *E*. *coli*, with an hourglass shape pore lumen. The constriction region is formed by the extracellular L3 loop with the narrowest diameter in the region about 5.5–6 Å, slightly narrower than the constriction region of OmpF [41]. While it has been generally accepted that these porins work as channels for small molecules of 600 Daltons or less, Ruggiu et. al. have demonstrated that even the molecules larger than the molecular weight of 600 Daltons can pass through these porins depending on their three-dimensional conformation, projection area, and dipole moment [46].

There could be several reasons of how and why biotinylation helps in the enhanced penetration through OmpC pore. One reason could be that biotinylation changes the molecular conformation and/or charge distribution in such a way that favors the passage through the OmpC pore. Further, attachment of biotin through an alkyl linker neutralizes the negative charge in the carboxylic acid group, increasing the net positive charge in the molecule, making it more favorable to pass through OmpC, which prefers cationic substrates. Our DFT calculations reveal regions of charge in the biotin tail, which may contribute to interactions with the ionizable residues in the pore lumen of OmpC and facilitate the molecule to slip through the pore.

OmpF and OmpC are highly similar non-specific porins. Kojima and Nikaido highlighted the major difference in the pore lumen of OmpF and OmpC [43]. There are 10 major ionizable residues which differ between OmpF and OmpC, namely D18, V29, N67, E68, W72, D135, D171, L173, R246 and K317 [43]. We picked three out of them, which were D18, W72 and D171 at three very different locations in the pore lumen. We found that when we mutate W72 into Lys and D171 into Thr, the corresponding amino acids present in OmpF, the accumulation of Atto565-biotin was reduced, suggesting that these ionizable residues might be involved in interactions with Atto565-biotin.

Recently, Prajapati et al. studied the translocation pathways of two fluoroquinolones, ciprofloxacin and norfloxacin, through OmpC, using molecular dynamics simulation with metadynamics and string method [47,48]. They observed the presence of interactions including salt bridges, hydrogen bonds, π-stacking, and hydrophobic contacts. It was observed that these fluoroquinolones orient differently when they are present at the beginning of the pore versus when they are in the constriction region depending upon the interactions involved. Also, even a small difference in structure between ciprofloxacin and enrofloxacin had a significant change in how they orient and with what residues in the pore they interact. They also observed that because of these differences in interactions, enrofloxacin translocation is associated with a higher energy barrier, which is in line with the experimental observation of slower diffusion of enrofloxacin than ciprofloxacin [49]. Relating this study to our observation, we might assume a significant difference in the interactions and orientations during the translocation of Atto565 versus Atto565-biotin. However, further studies involving the molecular dynamics simulation is required to shed light on the exact mechanism.

## Concluding remarks

In this study, we examined the effect of biotinylation on the accumulation of a fluorescent compounds, Atto565, in *E*. *coli*. We found that biotinylation indeed promote accumulation of Atto565, but not through the specific biotin-specific uptake system as we had expected. We found a two-fold increase of accumulation in the periplasm upon biotinylation. Several antibacterial targets have been explored in the periplasmic space of gram-negative bacteria and because of their location, antibacterial molecules need to cross only the outer membrane compared to both outer and inner membrane for cytoplasmic targets [2]. Hence, development of strategies that allow for the better penetration of molecules through the outer membrane are helpful in designing new antibacterial therapeutics. On a positive note, Sauvageot et al. reported that in the absence of photo illumination, biotinylation reduced the MIC of an Irridium (III) dipyridylamine complex by 8-fold in *Pseudomonas aeruginosa* [49]. No accumulation data was reported, thus it was not clear if the enhancement was due to accumulation. Further experimental, and molecular dynamics simulations would be necessary to understand the detail mechanism of this enhanced penetration of molecules through biotin conjugation.

## Supporting information

S1 Fig Florescence intensities of Atto565 and its conjugates showed conjugation did not significantly affect the florescence quantum yield. (DOCX)

S2 Fig Representative florescence intensities of Atto565 and its conjugates are linear with the concentration range used. (DOCX)

S1 Table Primers used for introducing the mutations in OmpC. (DOCX)

S1 Graphical abstract (TIF)
