# Supplementary material for: Biotinylation as a tool to enhance the uptake of small molecules in Gram-negative bacteria
Source: PLoS One. 2021 Nov 12;16(11):e0260023. doi: 10.1371/journal.pone.0260023 (PMC8589159; doi:10.1371/journal.pone.0260023)
Supplement: S1 Fig — (DOCX) [file pone.0260023.s001.docx]

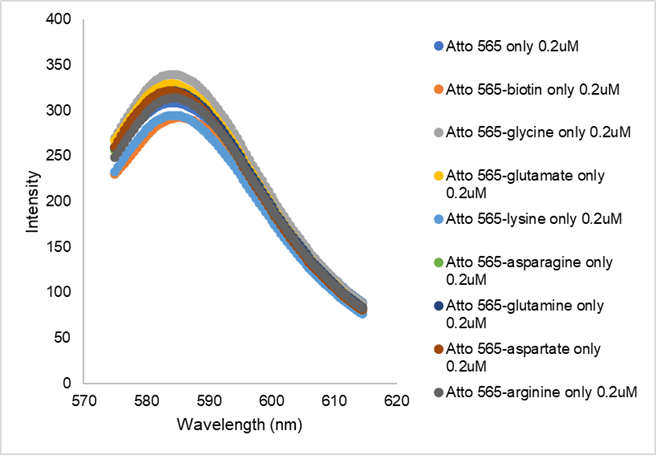


**S1 Fig.** Florescence intensities of Atto565 and its conjugates showed conjugation did not significantly affect the florescence quantum yield.
