# Supplementary material for: Biotinylation as a tool to enhance the uptake of small molecules in Gram-negative bacteria
Source: PLoS One. 2021 Nov 12;16(11):e0260023. doi: 10.1371/journal.pone.0260023 (PMC8589159; doi:10.1371/journal.pone.0260023)
Supplement: S1 Table — (DOCX) [file pone.0260023.s003.docx]

**S1 Table.** Primers used for introducing the mutations in OmpC.

| Mutation | Forward primer | Reverse primer |
| --- | --- | --- |
| D18V | 5’-CGGTAAAGTAGTCGGCCTGCAC-3’ | 5'- GTGCAGGCCGACTACTTTACCG -3' |
| W72K | 5’- CGAAAACAACTCCAAGACCCGTGTGGC -3’ | 5'- GCCACACGGGTCTTGGAGTTGTTTTCG -3' |
| D171T | 5’- CTAACAACGGTCGTACCGCACTGCGTC -3’ | 5'- GACGCAGTGCGGTACGACCGTTGTTAG -3' |
