# Supplementary figures and images for: Biotinylation as a tool to enhance the uptake of small molecules in Gram-negative bacteria
Source: PLoS One. 2021 Nov 12;16(11):e0260023. doi: 10.1371/journal.pone.0260023 (PMC8589159; doi:10.1371/journal.pone.0260023)

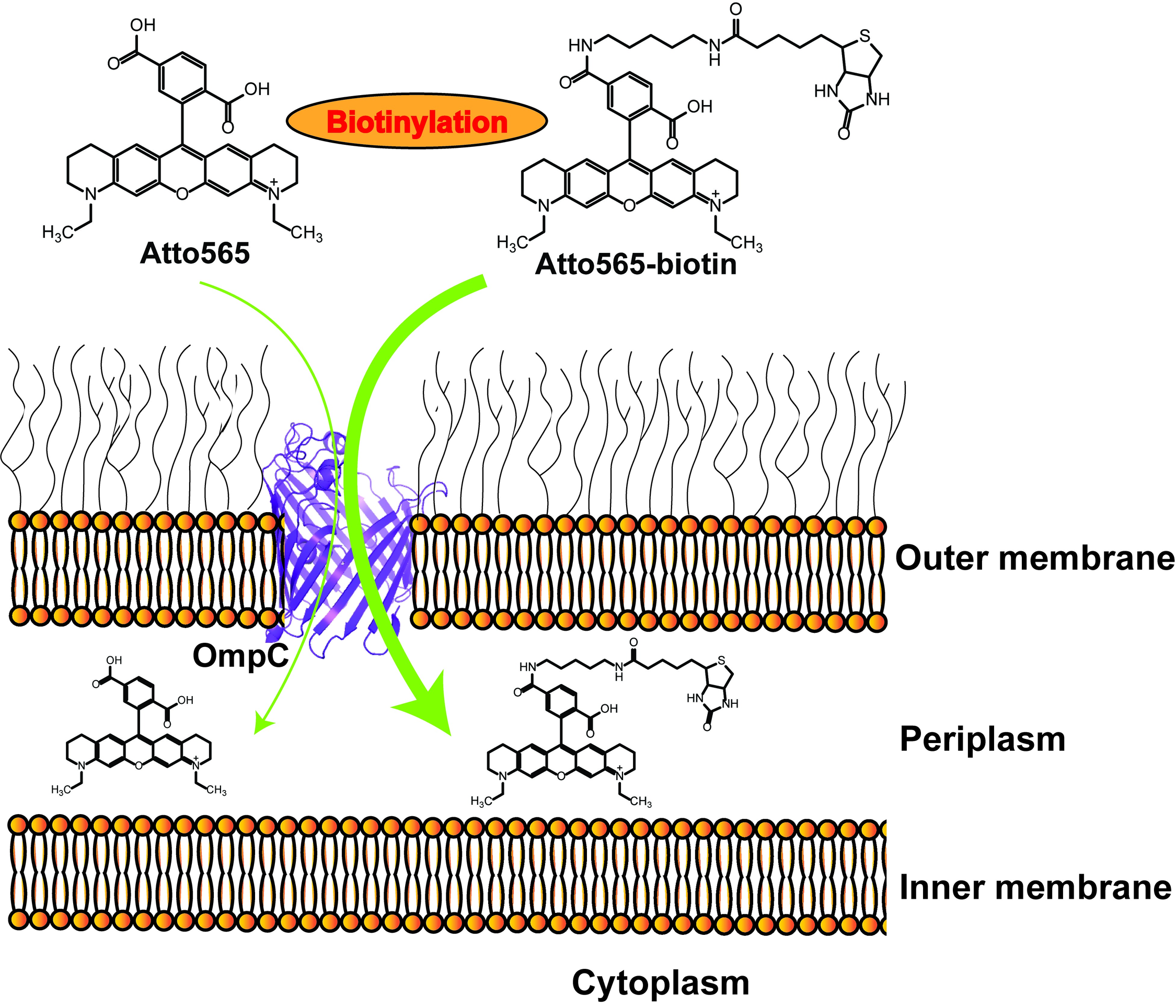

Supplement: S1 Graphical abstract — (TIF) [file pone.0260023.s004.tif]
